# Effect of Eyeglasses on Student Academic Performance: What Matters? Evidence from a Randomized Controlled Trial in China

**DOI:** 10.3390/ijerph191710923

**Published:** 2022-09-01

**Authors:** Kang Du, Huan Wang, Yue Ma, Hongyu Guan, Scott Rozelle

**Affiliations:** 1 College of Economics, Xi’an University of Finance and Economics, Xi’an 710100, China; 2 Center for Experimental Economics in Education, Shaanxi Normal University, Xi’an 710119, China; 3 Stanford Center on China’s Economy and Institution, Stanford University, Stanford, CA 94305, USA

**Keywords:** randomized controlled trial, eyeglasses, effectiveness, academic performance

## Abstract

Although eyeglasses have been considered a cost-effective way to combat myopia, the empirical evidence of its impacts on improving learning outcomes is inconsistent. This paper provides empirical evidence examining the effect of providing eyeglasses on academic performance between provinces with a different economic level in western China. Overall, we find a significant impact in Intention-to-Treat analysis and a large and significant local average treatment effect of providing free eyeglasses to students in the poor province but not in the other. The difference in impact between the two provinces is not a matter of experimental design, implementation, or partial compliance. Instead, we find that the lack of impact in the wealthier provinces is mainly due to less blackboard usage in class and wealthier households. Our study found that providing free eyeglasses to disadvantaged groups boosted their academic performance more than to their counterparts.

## 1. Introduction

Myopia poses a serious problem for students in China and may constrain their academic development if left uncorrected. A report in *Nature* detailed a “Myopia Boom” occurring in China, claiming that up to 90% of teenagers and young adults are myopic [1]. Mostly, children’s poor vision can be improved with properly fitted eyeglasses [2], but approximately 85% of myopic children in rural China are left uncorrected [3]. Uncorrected myopia in school-aged children, like other health disorders, may have adverse long-term consequences, like harming both school performance and attainment [4]. In response, the Chinese government launched an anti-myopia program which aimed to reduce the myopia rate of adolescents by a range of actions, such as increasing outdoor activities, reducing academic burdens, and providing timely corrective treatment [5].

It has been theorized that eyeglasses, by combatting myopia, can also improve myopic students’ academic performance by enabling them to see learning materials more clearly, such as the blackboard [6,7]. However, the existing empirical literature has shown mixed evidence about its effect on the academic performance of myopic students. While several randomized controlled trials (RCTs) in China found that providing eyeglasses improved myopic children’s performance in both primary and middle schools [8,9,10], the same intervention did not always improve academic performance. There are two studies discussing the heterogeneous treatment impacts of providing eyeglasses on academic performance. Study has found that despite providing free eyeglasses having a positive effect on student test scores on average (by 0.16–0.22 standard deviations), its impact varied even between counties [7]. Likewise, a recent paper compared two RCTs carried out in the east and west of China, respectively, and found that while providing free eyeglasses improves math test scores of myopic students by 0.14 standard deviations in provinces of western China, it has no impact in eastern areas [11].

Although these RCT evaluations overcome the issue of causality, efforts to understand why the impact of eyeglasses on student academic performance might vary across different individuals or regions have been limited. This variation is potentially important for designing future vision care programs and relevant policies. Therefore, more research to understand the heterogeneous impacts of providing eyeglasses on student academic performance is needed.

There are three potential explanations for the mixed evidence about the impact of eyeglasses on academic performance. Firstly, implementation problems may occur when providing and distributing eyeglasses. For example, one study mentioned that during the distribution of eyeglasses at the RCT, students in the control group were mistakenly provided with eyeglasses, which may in part have undermined the empirical evidence [7]. Secondly, partial compliance may also threaten the evaluation of RCTs [12]. Specifically, in many similar programs, some students or parents in the treatment group refused free eyeglasses due to the belief that wearing them worsens vision problems, which poses a partial compliance problem [13]. Thirdly, while an RCT can yield an unbiased estimate, its impact across samples from different contexts may vary [14,15]. Previous study found that providing eyeglasses significantly improves students’ math scores in rural public schools but not in private migrant schools due to the difference in schooling contexts [11]. Similar evidence is found in other areas. For example, a school-based computer-assisted learning program shows that students from poor families benefited more [16]. In the programs providing parental stimulation to infants and toddlers, children with lower levels of initial skills at baseline benefited the most from the interventions [17]. Therefore, it is possible that students who are already disadvantaged or living in poorer regions may experience more of the benefits of eyeglasses than students from more well-off backgrounds.

In this paper, we take advantage of a well-implemented RCT and collect measures of compliance and other relevant features, including the characteristics of individuals and their families in different contexts, through our survey. Run in two different provinces, our RCTs followed the same protocols and were carried out in the same period. Though the two provinces are adjoint in geography, the sample city selected varies widely in socioeconomic status. This feature allows us to examine whether the eyeglasses intervention was more or less effective with different backgrounds as well as the conditions in which eyeglasses may or may not be effective in improving academic performance.

The goal of this paper is to (1) explore suitable conditions where providing eyeglasses is an effective tool in improving academic performance and (2) to analyze why the same programs worked or not by testing two hypotheses: partial compliance and difference in sample backgrounds. We reach that goal through the following steps. First, we measure the average impact of providing eyeglasses on student academic performance. Second, we examine whether the impact varies across different regions. Third, we examine the above two hypotheses that seek to explain why the impact of providing eyeglasses might differ between the two regions.

The remainder of the paper is organized as follows. Section 2 introduces the research setting. Section 3 describes the sampling, data collection, experimental design, balance and attrition, and statistical approach. Section 4 details the results. Section 5 presents our discussions and conclusions.

## 2. Materials and Methods

### 2.1. Study Area and Sampling

We conducted a randomized controlled trial in rural areas of two provinces—Gansu and Shaanxi—in western China. The per capita gross domestic product in Shaanxi was USD 6108, which is similar to that of the national average. In contrast, the GDP per capita in Gansu was USD 3976, which is the third-poorest province in China.

The experiment was implemented in primary schools located in the rural county areas of Tianshui city for Gansu and Yulin city for Shaanxi. Both cities have a population of about 3.3 million, which accounts for 10% of the population in each province, but they differ in terms of economic development. Tianshui, with a GDP per capita of USD 2680, is relatively similar to its provincial average level [18]; Yulin, with a GDP per capita of USD 13,100, is higher than its provincial average level, which is partially due to its mining-related industries [19]. As the two cities’ income levels differ, their selection as sample sites allows us to investigate whether the identical vision care program could have distinct impacts in rural China regions with varying socioeconomic status.

To select our sample schools, we used official records from all county education bureaus to obtain a list of all rural primary schools in the two cities, excluding one county in Yulin owing to its small population size. We limited our selection of schools to those with between 50 and 150 children in the fourth and fifth grades combined for implementation efficiency and logistical considerations. To avoid possible spillovers, one (and only one) school at random from each township in each county was chosen in the sample frame.

After selecting the sample schools, one class from each of the fourth and fifth grades was randomly chosen from each sample school. Students in the fourth and fifth classes were selected because, according to previous studies conducted in rural China, myopia typically occurs between the ages of eight and ten, which corresponds to the fourth and fifth grades [20]. Finally, 6938 students enrolled from 80 schools in Gansu and 6422 students enrolled from 88 schools in Shaanxi were interviewed.

### 2.2. Experimental Design

We randomly allocated the sample schools to either a treatment or a control group in October 2012, following the baseline survey and a two-step eye test (details in Section 2.3.1). To ensure a balanced sample and to boost the power of the experimental design, we stratified the intervention assignment by location (county), school size, and eye examination results. Half of the schools in each stratum were randomly allocated to the treatment group and the other half to the control group. In total, our study included 167 schools with a minimum of 10 students for each school and an intraclass coefficient of 0.10. Assuming an α of 0.05 and an R^2^ of 0.5, our study was powered to observe an effect size of 0.20 standard deviations at 90% power. The experimental design was concealed from the students and their families.

After the sample assignments, the research team conducted the intervention in the treatment schools. To do so, every treatment school student was screened. After that, those students that were flagged as possibly having vision problems were given an additional refraction examination. If a student was diagnosed with myopia by the program optometrist, he/she was then presented with a prescription that could be used to fit a free pair of eyeglasses. A letter about myopia status and an eyeglass prescription were also sent to students’ parents. At the same time, a similar protocol was followed in the control schools. After screening and conducting eye examinations, a letter containing a prescription was given to the parents of myopic students.

The only difference between control schools and treatment schools was that free eyeglasses were not dispensed in control schools. In contrast, program optometrists revisited the schools and dispensed free eyeglasses in the treatment schools four weeks after the baseline survey. The trial was approved by Stanford University (No. ISRCTN03252665, registration site: http://isrctn.org, accessed on 4 August 2018).

### 2.3. Data Collection

#### 2.3.1. Baseline Survey

Before implementing the intervention, the baseline survey was organized in September 2012. During the baseline survey, the enumeration team collected comprehensive information on all students, their parents and school teachers through separate questionnaires.

The student survey collected two types of information. In the first block, enumerators administered questionnaires to students about their individual and family characteristics. Individual characteristics contain student age, grade, gender, and whether they were wearing eyeglasses prior to the intervention and after school activities. Family characteristics include their parents’ education and migration status.

In the second block, we included a standardized math scale that was designed to measure student learning outcomes. The research team produced separate mathematics tests that were appropriate for children in grades 4 to 5. With the support of local educators, questions for these tests were chosen from the Trends in International Mathematics and Science Study. The test was timed (25 min) and proctored by two enumerators in each school. For analyses, we normalized scores using the control group’s baseline mean and standard deviation as the basis for the normalization.

We also administered questionnaires to parents and teachers; 13 survey questions were given to parents to answer on their ownership of various products, such as televisions, washing machines, cameras, etc. Answers to these questions were used to estimate value of household assets. In addition, teachers in the sample classes were asked questions about their professional status, educational background, the share of course curriculum material that was taught on the blackboard and the overall size of their class.

#### 2.3.2. Eye Examination

All treatment and control students underwent a two-step eye examination as part of the baseline survey. The eye examination teams were trained by the Zhongshan Ophthalmic Center (ZOC) at Sun Yat-sen University. An initial screening of students’ visual acuity (VA) using the Early Treatment Diabetic Retinopathy Study (ETDRS) eye charts was conducted by a team of two trained staff [21]. Participants in the second-step vision test were those who failed the vision screening (the standard for which is defined by VA ≤ 6/12 in either eye). We measured and compared different levels of VA by LogMAR, one of the most commonly used continuous scales in the field of ophthalmology [22]. When interpreting the results, it is important to remember that the higher the LogMAR value, the worse one’s vision is. As a second step, the refraction test was then performed by a team of three people, comprising an optometrist, a nurse, and an assistant. To estimate prescriptions for children’s eyeglasses, the team used cycloplegic automated refraction with subjective refinement (the cutoff for myopia is 0.75 D).

#### 2.3.3. Endline Survey and Compliance Check

In May 2013, near the end of the 2012–2013 academic year and approximately seven months after the baseline survey, we conducted the endline survey. The instruments for this survey were similar to the baseline survey. To collect post-treatment math test scores, the team administered a 25-min math test to all students in the control and intervention groups.

To check the compliance of the experiment, a group of two enumerators went to the sampled schools and conducted unannounced checks before the endline survey. The enumerators first obtained a list of the students who were diagnosed as myopic and received free eyeglasses at the baseline. Then, they stood outside of the classrooms to record individual-level information on eyeglass wearing.

The experiments in the two provinces are particularly comparable. For both baseline and endline, we followed the same protocols in Gansu and Shaanxi. Specifically, we conducted the same surveys, used the same standardized mathematics exams and measured compliance in the same way. The research teams that implemented both surveys were the same.

### 2.4. Balance and Attrition Check

Of the 13,360 students in the 168 sample schools who received an eye exam at baseline, 2169 were found to need eyeglasses and 2087 were followed up at the endline. Only those students who were followed up were included in the analysis sample, which resulted in a total of 762 students in Gansu and 1325 students in Shaanxi.

Overall, the baseline characteristics were well balanced among each province’s control and treatment groups. Baseline characteristics of the two provinces across the treatment and control groups are shown in Table 1a. Columns 3 and 6 of Table 1a show the differences of each of the baseline characteristics between the treatment and control groups in Gansu and Shaanxi, respectively. Statistical significance of mean differences is estimated by regressing each of the baseline characteristics on the treatment dummy (treatment group = 1; control group = 0). The only significant difference between any of the characteristics was the share of teachers that held credentials, which was slightly higher in the control group than in the treatment group in Gansu. This difference is significant at the 10% level. None of the characteristics was statistically different across the treatment and control groups in Shaanxi.

Table 1b shows that there was only one statistically significant difference in terms of baseline characteristics between the treatment and control groups when pooling the two provinces together. The share of teachers that held credentials was significantly higher in the control group at the 5% level.

The attrition rate of our study was low, according to relevant RCT literature [23]. Specifically, only 24 (2.9%) out of 802 students in Gansu and 35 (4.2%) out of 1387 students in Shaanxi could not be followed up. To examine if attrition affected our results, we checked for the balance between “missing” and “non-missing” subsamples. We ran this balancing test by regressing each student’s baseline characteristics on the treatment dummy and clustering the standard errors at the school level. None of the coefficients are statistically significant, showing that student attrition is independent of treatment assignment in both the pooled sample and each of the regional samples, according to Appendix A, Table A1.

### 2.5. Statistical Approach

In this paper, we conducted three types of analysis. We first used the Intention-to-Treat (ITT) analysis to measure the overall effect of the program regardless of compliance. The ITT effect captures the impact of being provided with eyeglasses, which is of immense interest to policymakers [24]. However, since not all myopic students wore the eyeglasses provided by the program (partial compliance), we also estimated results from the Local Average Treatment Effect (LATE). The LATE scales up the treatment to take account of partial compliance and reveals the actual impact of wearing eyeglasses [12,24]. Lastly, we estimated the interaction terms of the intervention and baseline characteristics to analyze the heterogeneous treatment impacts of eyeglasses. All analyses were performed using STATA 15.1 (Stata Corp., College Station, TX, USA).

#### 2.5.1. ITT Effect

We used the ordinary least squares (OLS) regression to estimate the impact of providing eyeglasses on our outcome variables via two methods.

First, we used an unadjusted model:

(1) $$y_{1i}=\beta_{0}+\beta_{1}Z_{i}+\beta_{2}y_{0i}+\varepsilon_{i}$$

where $y_{1i}$ is the standardized math scores for student i at the endline, $Z_{i}$ is a treatment dummy that takes the value of one if the student i was assigned to the treatment group, and $y_{0i}$ is the standardized math score at the baseline. $\varepsilon_{i}$ is the error term clustering at the school level.

Next, we conducted an adjusted analysis with more statistical power by controlling for all baseline characteristics shown in Table 1 and strata fixed effects. The adjusted model is specified as:

(2) $$y_{1i}=\beta_{0}+\beta_{1}Z_{i}+\beta_{2}y_{0i}+{X^{\prime }}_{i}\gamma +\varphi_{s}+\varepsilon_{i}$$

where the variables and parameters are the same as those in Equation (1) with the inclusion of an extra set of baseline characteristics ${X^{\prime }}_{i}$ and strata fixed effects $\varphi_{s}$. The matrix ${X^{\prime }}_{i}$ contains 12 student, family, teacher, and school control variables. These variables are often used as control variables in studies that identify the effect of providing eyeglasses on academic performance [10,11]. Specifically, of the student characteristics, we controlled for grade, gender, visual acuity, eyeglasses wear, and baseline math score. Of the family characteristics, we controlled for parental education, parental migration status, and family economic status. For teacher and school characteristics, we controlled for class size, professional status of teachers, and their educational background. In addition, the main role of using eyeglasses is to help students see the blackboard [7], and the frequency of students’ use of eyeglasses is influenced by the frequency of the teachers’ use of the blackboard. Therefore, we also controlled the usage of the blackboard in class. In both models, the parameter $\beta_{1}$ measured the ITT effect.

#### 2.5.2. LATE Effect

Despite that RCT is the gold standard for impact evaluation, the above ITT estimate might not accurately reflect the treatment effects because of partial compliance. In our case, many students, parents, and teachers have a misunderstanding of the importance (and safeness) of wearing eyeglasses (when children have vision problems), which may lead to partial compliance [13]. Partial compliance is commonly found in eyeglass promotion programs [25,26]. To test this, researchers have used an instrumental variable approach to estimate the LATE version of the impact. The LATE coefficient measures the effect of the treatment on those who actually complied with the intervention in the treatment group [24]. Here, the endogenous variable is wearing eyeglasses (i.e., the actual treatment status), and the instrumental variable is providing eyeglasses (i.e., the initial random assignment of the treatment status).

To capture the impact of wearing eyeglasses, the LATE model formulation substitutes the $Z_{i}$ with $T_{i}$ in Equation (2). Specifically, the LATE model is:

(3) $$y_{1i}=\beta_{0}+\beta_{1}T_{i}+\beta_{2}y_{0i}+{X^{\prime }}_{i}\gamma +\varphi_{s}+\varepsilon_{i}$$

where $T_{i}$ is a dummy variable that equals one if a student i actually wore eyeglasses and zero if the student did not. As $T_{i}$ might be associated with the unobservable factors, we use the variable $Z_{i}$ to instrument $T_{i}$. Thanks to the random treatment assignment of $Z_{i}\;\mathrm{and}\;\mathrm{its}\;$ strong predictive power for $T_{i}$, $Z_{i}$ is uncorrelated with the error term $\varepsilon_{i}$. Therefore, the parameter $\beta_{1}$ in Equation (3) measures the LATE effect.

#### 2.5.3. Heterogeneous Effects

To see whether the impact of providing eyeglasses is significantly different between the two provinces and whether the impact varies among different types of students, we used the following equation to analyze the heterogeneous impact of the intervention:

(4) $$y_{1i}=\beta_{0}+\beta_{1}Z_{i}+\beta_{2}y_{0i}+\beta_{3}P_{i}+\beta_{4}Z_{i}P_{i}+{X^{\prime }}_{i}\gamma +\varphi_{s}+\varepsilon_{i}$$

where $P_{i}$ is a dummy variable representing the two provinces, and is equal to one if student i lives in Gansu and zero otherwise. In this model, $\beta_{1}$ measures the impact of providing eyeglasses on students who live in Shaanxi province, and $\beta_{1}+\beta_{4}\;$ is the impact on students who live in Gansu province. The coefficient $\beta_{4}$ measures the difference in impact of providing eyeglasses between the two provinces.

## 3. Results

### 3.1. The Impact of Providing Eyeglasses on Academic Performance (Intention-to-Treat, ITT)

Table 2 reports the effect of providing eyeglasses on academic performance using the ITT model. When we examine the results using the pooled sample from both provinces, the results show a significant and positive ITT effect on the standardized math test scores of myopic students. Compared to the control group, providing subsidized eyeglasses improved the average test score of students with myopia by 0.060 standard deviations using the unadjusted model (Column 1). The coefficient increases to 0.062 standard deviations when controlling for all baseline covariates (Column 2). These estimates are both statistically significant at the 1% level.

Furthermore, we examined whether the interventions had different effect sizes between the two provinces. Our heterogeneous results showed that the ITT effects in Shaanxi were fundamentally different from those in Gansu. The interaction estimate, which measures the difference in the impact of providing eyeglasses between the two provinces, was statistically significant at the 5% level (Row 3). This indicates that behind the average effect of the full sample exists substantial heterogeneity between the two provinces.

Specifically, we only found a significant ITT effect in the schools from Gansu but not in the schools from Shaanxi. In Gansu, when using the unadjusted model, providing free eyeglasses improved myopic students‘ standardized math scores by 0.122 standard deviations compared to the control group (Row 4, Column 3), which is statistically significant at the 1% level. According to the adjusted model, the magnitude of the coefficient is very similar, i.e., 0.123 standard deviations with statistically significant at the 1% level (Row 4, Column 4). In Shaanxi, however, the point estimates are smaller and standard errors are large in both the unadjusted and adjusted models, resulting in no significant impact in either model in Shaanxi (Row 5).

### 3.2. Potential Mechanism of Differential Effect between Two Provinces

What factors lead to the disparities in the treatment effect between the two provinces? Our analysis so far has shown a significant difference in the ITT effect between the two provinces. Given the discussion in an earlier section (i.e., the program was well implemented and the same procedure was followed in both provinces throughout our study), in this section, we will try to examine why the results differ in the two provinces. To do so, in the next two subsections, we will examine two hypotheses. We firstly examine the Hypothesis 1:

**Hypothesis** **1.** *Partial compliance is the reason for the lack of significant impact in Shaanxi.*

In the second subsection, we test the Hypothesis 2:

**Hypothesis** **2.** *The difference in impact was due to students in Gansu having certain background characteristics that made them benefit more from the eyeglasses.*

### 3.3. Hypothesis 1: Partial Compliance

To explain disparities in the results, we surmised that the compliance rate might be low in Shaanxi province relative to Gansu province and that this low compliance undermined the impact of the intervention. Specifically, we tested whether the difference in the ITT of providing eyeglasses was driven by the fact that the myopic students (for some reason) were less likely to wear eyeglasses in Shaanxi than those in Gansu.

#### 3.3.1. Compliance

To test this hypothesis, we compared the treatment and control groups’ compliance with wearing eyeglasses at endline (Table 3). In both provinces, the proportions of myopic students who wore eyeglasses at baseline were evenly distributed across treatment and control groups. At the endline, we found that about 38.4% of the students were wearing eyeglasses in Gansu, and this number was 41.6% in Shaanxi. Considering that we gave all nearsighted students in the treatment group a pair of eyeglasses, partial compliance existed in both provinces. However, the shares of myopic students who wore their glasses at endline were significantly increased compared to the control groups in both provinces.

Given that overall compliance was low, the next step then is to examine whether the intervention had a different impact on wearing eyeglass between the two provinces. To do so, we will determine whether partial compliance was different between the two provinces.

The differences in compliance between the two provinces are shown in Table 4. According to the results, providing free eyeglasses improved the share of wearing eyeglasses by about 14 percentage points overall (Columns 1 and 2). The increase in wearing eyeglasses post-intervention was 15.1 percentage points in Gansu and 13.2 percentage points in Shaanxi (Column 4, Row 5 and 6). Although the share is slightly higher in Gansu (1.9 percentage points), the impact of the intervention on the wearing of eyeglasses is not significantly different between the two provinces (Row 4). This implies that partial compliance fails to explain the significant disparities of ITT impacts between schools in Gansu and schools in Shaanxi.

#### 3.3.2. Impact of Wearing Eyeglasses (Local Average Treatment Effect, LATE)

Though partial compliance may not be the reason for disparities in ITT impacts between the two provinces, we further analyzed the impact of wearing glasses by using the LATE model (Table 5), which takes partial compliance into account. In the LATE model, it is necessary to test whether our instrumental variable is weak or not [27]. The F test values of the first stage regressions were all well above 10 (Row 5), which indicates weak IV is not a problem in this study.

The results in Columns 1 and 2 suggest that wearing eyeglasses improved standardized math test scores by 0.861 to 0.918 SD in the pooled sample compared to control schools. Yet, when we ran the same regressions in the two subsamples, just like the results for the ITT model, we still only found a significant impact in Gansu and not in Shaanxi. Controlling baseline variables, wearing eyeglasses improved the standardized math score of myopic students in Gansu by 1.770 SD compared to the control group, which is significant at the 1% level (Table 5, Column 4). However, regardless of whether we use the unadjusted or adjusted LATE models, we detect no significant impact in Shaanxi. Results under the LATE model provide further evidence that partial compliance might play, at most, a limited or non-existent role that leads to the variations in impacts.

### 3.4. Hypothesis 2: Difference in Sample Backgrounds

Partial compliance, as discussed above, is not the cause of differences in impact between two provinces. It might be possible that the difference in impacts of providing eyeglasses stems from the differences in sample backgrounds. Students with more disadvantaged backgrounds may derive more benefits from eyeglass interventions. A related study showed that the benefits of providing eyeglasses are greater for under-performing students [7]. In our study, if the backgrounds of students in Gansu were more disadvantaged than those in Shaanxi, it is plausible that the intervention had a larger effect on students in Gansu.

#### 3.4.1. Difference in Baseline Characteristics

In Table 6, we compare all baseline characteristics shown in Table 1 between the two provinces. We found that five variables were significantly different between the two provinces when comparing the baseline characteristics.

First, more myopic students in Gansu did not have eyeglasses at baseline. Specifically, the share of students wearing eyeglasses was four percentage points in Gansu lower than those in Shaanxi (*p*-value < 0.1). Second, students in Gansu scored lower than students in Shaanxi by 0.09 standard deviations at the baseline standardized math score (*p*-value < 0.05). Third, parents in Gansu out-migrated for work more frequently than those in Shaanxi. About 52.4% of students in Gansu had at least one migrant parent compared to 42.8% in Shaanxi (*p*-value < 0.05). Fourth, student families in Gansu were much poorer. On average, a household’s assets in Gansu (14,800 RMB) was about 2.7 times lower than that in Shaanxi (40,000 RMB). Fifth, 83.1% of teachers in Gansu used the blackboard to teach more than half of the curriculum, while only 56.5% of teachers in Shaanxi did the same.

According to these five indicators, there are significant differences between Gansu and Shaanxi, especially in economic status. The relationship between household wealth and academic achievement is well acknowledged in the education literature [28]. As one might expect, wealthier parents can provide more and better learning opportunities for their children, such as buying books, school kits and obtaining access to tutoring if needed [29]. Thus, it is unsurprising that more students already had eyeglasses and had a higher math score in Shaanxi at baseline. Furthermore, a study showed that the poor are more likely to migrate to the cities to seek work [30]. As we can see in the above comparison, more parents out-migrate in Gansu. Lastly, although the quality of teachers is almost the same between the two provinces, schools in Shaanxi might enjoy better facilities due to the higher economic status. Students’ average educational expenditure in Shaanxi (3343 RMB) is about two times higher than that in Gansu (1585 RMB) during elementary school [18]. Such an advantage may allow teachers more hardware and software teaching resources, lending teachers more flexibility beyond using traditional blackboards. Hence, although these five variables are distinct, they all relate to economic status to some extent.

#### 3.4.2. Heterogeneous Effect on the Subgroup with Differential Characteristics

The differences in the above five characteristics indicate that students in Gansu are disadvantaged compared to their counterparts in Shaanxi, especially in economic status. If the intervention has a different effect on the subgroup of students with those five characteristics, the differences in the observed impacts may be explained by the differences in the baseline characteristics across the two provinces. In the rest of this subsection, we pool the sample together and conduct a heterogeneity analysis to investigate whether the difference in treatment effects across the two provinces can be explained by the five baseline characteristics described above (Table 7). For convenience, we define a dummy variable to replace standardized math scores. We take its value as one if the baseline math score is below the average and zero otherwise. Similarly, we also define a dummy variable to replace household asset value and take its value as one if a household asset score is below the average and zero otherwise. We also present the difference of those two dummy variables between the two provinces in Table 6 (Rows 6 and 10).

Table 7 presents the heterogeneity results of each of the five variables discussed above. In each column, the positive interaction term suggests that the impact of providing eyeglasses on math scores is higher for myopic students with more disadvantaged backgrounds. However, we find weaker evidence that providing eyeglasses had a larger effect on children without eyeglasses at baseline (Column 5). The program increased the math score of children without eyeglasses at baseline by 0.062 SD but had no detectable effect on those counterparties. This difference, however, was not significant.

#### 3.4.3. The Differential ITT Impact Explained by Hypothesis 2

Additionally, we try to determine the extent to which the four variables with significant interaction terms may be responsible for the observed difference in program impacts between the two provinces (Table 8). Specifically, these variables include: at least half of class material was taught on the blackboard, below-average math score, below-average household asset score, and parental out-migration.

The findings in Column 4 of Table 2 reveal that there is a total difference of 0.092 SD in the estimated program impact (the coefficient is 0.123 for Gansu and 0.031 for Shaanxi). Table 8, Column 1 illustrates how much of the difference in impact between both provinces can be explained by blackboard usage alone. As shown in Table 6, the share of teachers in Gansu that use the blackboard is 0.271 SD higher than in Shaanxi. The interaction coefficient of 0.100 is statistically significant at the 1% level (Table 7). Blackboard usage, when considered together, has a differential impact of 0.027 standard deviations (0.271 × 0.100) and contributes 29.45% of the overall impact difference of 0.092 SD (0.027/0.092). These results indicate that about 29.45% of the difference in impacts may be attributed to the difference in blackboard usage between the two provinces. Using the same method described above, we find that difference in math score accounts for 3.26% (0.003/0.092) of the total difference in impact (Column 2). The difference in household assets accounts for 35.87% (0.033/0.092) of the total difference in impact (Column 2), and parental out-migrating work explains 6.52% (0.006/0.092) of the total difference. Taken together, these four variables can explain about 75% of the difference in the impact of providing eyeglasses in Gansu and Shaanxi.

Based on these findings, we conclude that the absence of an impact cannot be attributed to partial compliance. In fact, the differences in the characteristics between the two provinces can account for most of the differences in program impacts.

## 4. Discussion

It is commonly believed that eyeglasses are a useful tool to combat myopia. However, the effect of eyeglasses on myopic students’ academic performance remains unclear. This paper examines the impact of providing eyeglasses on standardized math scores based on a well-designed randomized controlled trial in two different provinces (Shaanxi and Gansu). Meanwhile, we investigated whether or not the intervention was effective in both provinces and reasons for the differences.

Overall, we found that providing eyeglasses to myopic students raises their math scores significantly, but its impact differs between the two provinces. While our estimations show that providing myopic students with eyeglasses boosts their math results by 0.123 standard deviations in Gansu, we found no significant influence in Shaanxi (Intention-to-Treat effect, ITT). Considering that many students do not actually wear the eyeglasses after they receive them, we use two ways to examine whether partial compliance leads to the differential impacts of the intervention. By comparing the compliance rate between the two provinces, we find that the intervention has the same impact on eyeglass wearing. Moreover, in order to deal with partial compliance, we also estimate the effect of the treatment on the treated (Local Average Treatment Effect, LATE). We found a significant and positive impact of wearing eyeglasses on academic performance in Gansu. However, we still found no significant impact in Shaanxi with or without adjustment.

Our analysis suggests that the lack of a detectable impact of providing eyeglasses in Shaanxi is not due to experimental design, implementation, or absence of compliance. Instead, our findings give insight into the underlying processes responsible for the disparities in intervention impacts, i.e., differences in the two provinces’ characteristics. By comparing all student, family, teacher, and school characteristics, we found that 5 baseline variables are significantly different. Furthermore, we examined whether the intervention was more or less effective with different subgroups of those characteristics. Specifically, our results indicate that the interventions have a significantly greater impact on students who have a lower baseline score, are left-behind children, and come from families with less wealth. Students in schools that use more blackboards in teaching also benefit more from the intervention. Students with those four characteristics can be seen as more disadvantaged than their counterparts, and, compared to students in Shaanxi, a much higher proportion of students in Gansu fall into those subcategories. According to our calculation, those four variables account for 75% of the differences in impact, which may explain why the intervention has no significant impact in Shaanxi but in Gansu.

The relevant study may be useful to reference in support of our findings that the eyeglass intervention has no effect on raising students’ academic performance in Shaanxi. Another vision care program which aimed to improve students’ academic performance by providing eyeglasses also showed a larger impact among underachievers compared to the achievers [7]. Students in Shaanxi had 0.18 standard deviation higher baseline scores than students in Gansu. Thus, the baseline math score was too high to detect a large and significant impact in Shaanxi.

Additionally, we believe that the substitution effect of wealth also contributes to a different influence on the two provinces. As we have discussed in the Results section, all those differential characteristics are correlated with socioeconomic status, which agrees with the literature [31]. In our paper, even though the two provinces are adjoint in geography, the per capita GDP for our sample city selected from Shaanxi is nearly five times higher than that of its counterpart in Gansu (USD 13,100 vs. USD 2680). Wealthier parents in Shaanxi have the capacity to provide more and better learning opportunities for their children if needed [29]. For example, we found more students in Shaanxi already wearing eyeglasses at baseline and using a computer to learn after school. Studies also show that wealthier parents tend to be better educated [32]. As we found, fewer parents go out for work in Shaanxi, which indicates that parents may have more time and are more capable to tutor their children after school. Therefore, we believe that because students in Shaanxi have more access to better or alternative learning resources, the impact of improved vision through eyeglasses on academic performance is lessened, as their academic performance relies less on the ability to see learning materials at a distance. Last but not least, schools in Shaanxi might have more hardware and software resources of multimedia teaching, which give teachers more flexibility beyond only using traditional blackboards. Therefore, students might use eyeglasses less frequently in Shaanxi, leading to a smaller or less significant impact. Due to all of these factors, students in wealthier Shaanxi seem to have more ways to study (multimedia learning, after-school computer learning, and tutoring). Thus, the effect may be smaller since richer students in Shaanxi do not entirely rely on eyeglasses, the main use of which is to see the blackboard clearly in school and to learn.

We also acknowledge the main limitations of this study: we have no data to verify all the mechanisms of the substitution effect of wealth, such as multimedia learning. Despite this limitation, the results have important implications. Providing eye care, for example, eyeglasses, can improve academic performance on average and may help narrow the gaps in equalizing academic performance. Studies also show that providing eyeglasses is relatively cost-effective and safe when used for educational purposes [33]. Additionally, our study also demonstrates that the design and upscaling of interventions need to take full account of the importance of context in order to maximize the impact of the intervention.

Furthermore, our study not only gives more evidence about the effect of providing eyeglasses but also adds to previous studies on the conditions under which the interventions really work. If the goal of a non-governmental organization is to increase the learning of children and eliminate educational inequality by providing eyeglasses, then doing so in a poorer setting will be more productive. Furthermore, eyeglasses can be used as an educational tool to narrow the gap between the poor and the rich. Other school-based interventions aimed to improve students’ academic performance also show that students from poor families benefited more [16,34]. The government can provide subsidies to the poor so that their myopic children can wear eyeglasses in time. In a broader sense, the findings on the varied effects of the vision care programs point to the possibility that tailored solutions may be needed in order to achieve health policy objectives among sizable subpopulations.

## 5. Conclusions

Based on a randomized controlled trial, this study assesses the impact of providing eyeglasses on student academic performance across different provinces and analysis the reasons behind the differences in the impact size. We found that students from disadvantaged groups with poor areas, more blackboard use, lower academic performance, and parents migrated for work benefited more from providing eyeglasses. Although partial compliance was not the cause of the difference in intervention effects between the two provinces, compliance with wearing eyeglasses in our sample was low. The compliance rate in treatment schools was only 38.4% in Gansu and 41.6% in Shaanxi, respectively. In the future, vision programs need to focus on improving compliance. Our results show that if this can be accomplished, more students would benefit from eyeglasses, especially those who are disadvantaged. More research is needed to understand why compliance is low and how to improve it. Answers to these questions will improve the design of policies to help more students benefit from eyeglasses.

## Figures and Tables

**Table 1:** (**a**) Baseline characteristics difference between treatment and control groups by province. (**b**) Baseline characteristics difference between treatment and control groups using pooled sample.

*Panle (**a**) by Province*						
	**Gansu (*n* = 762)**			**Shaanxi (*n* = 1325)**		
**Variables**	**Control Group**	**Treatment Group**	**Difference**	**Control** **Group**	**Treatment Group**	**Difference**
	**(1)**	**(2)**	**(3)**	**(4)**	**(5)**	**(6)**
**Student:**						
Grade (1 = fourth)	4.607	4.637	0.03	4.604	4.58	−0.024
	(0.489)	(0.481)	(0.041)	(0.489)	(0.494)	(0.029)
Male sex (1 = yes)	0.509	0.525	0.016	0.498	0.472	−0.026
	(0.500)	(0.500)	(0.036)	(0.500)	(0.500)	(0.031)
LogMAR	0.501	0.489	−0.012	0.519	0.524	0.006
	(0.257)	(0.241)	(0.025)	(0.23)	(0.210)	(0.018)
Not wearing eyeglasses at baseline (1 = yes)	0.887	0.859	−0.028	0.834	0.83	−0.004
	(0.318)	(0.348)	(0.022)	(0.372)	(0.376)	(0.031)
Baseline standardized math score	0.137	0.149	−0.012	0.290	0.360	−0.070
	(0.100)	(0.945)	(0.070)	(1.007)	(0.974)	(0.055)
**Family:**						
One or both parents with ≥12 years of education (1 = Yes)	0.217	0.252	0.035	0.193	0.208	0.015
	(0.412)	(0.434)	(0.037)	(0.394)	(0.403)	(0.027)
Family asset value, 10 thousand	1.433	1.539	0.105	3.709	4.25	0.541
	(1.832)	(1.963)	(0.136)	(3.547)	(3.912)	(0.435)
One or both parents out-migrated for work (1 = yes)	0.517	0.53	0.013	0.438	0.419	−0.019
	(0.500)	(0.5)	(0.049)	(0.497)	(0.494)	(0.042)
**Teacher & Schools:**						
Teachers finished college or higher education	0.478	0.496	0.019	0.516	0.521	0.005
	(0.500)	(0.500)	(0.100)	(0.500)	(0.500)	(0.095)
Share of teachers that held professional titles	0.989	0.836	−0.153 *	0.917	0.853	−0.064
	(0.429)	(0.212)	(0.073)	(0.134)	(0.192)	(0.038)
Class size	47.358	47.021	−0.338	42.286	44.284	1.998
	(10.281)	(11.196)	(2.437)	(11.738)	(13.133)	(3.034)
Half and above material taught on blackboard (1 = yes)	0.879	0.783	−0.095	0.638	0.501	−0.137
	(0.327)	(0.413)	(0.079)	(0.481)	(0.500)	(0097)
Number of observations	379	383	-	609	716	-
*Panel (**b**) Pooled Sample*						
	**Pooled Sample (*n* = 2087)**					
**Variables**	**Control Group**		**Treatment Group**		**Difference**	
	**(1)**		**(2)**		**(3)**	
**Student:**						
Grade (1 = fourth)	4.605		4.600		−0.005	
	(0.489)		(0.49)		(0.024)	
Male sex (1 = yes)	0.502		0.490		−0.012	
	(0.500)		(0.500)		(0.024)	
LogMAR	0.512		0.512		0.000	
	(0.241)		(0.222)		(0.015)	
Not wearing eyeglasses at baseline (1 = yes)	0.854		0.840		−0.014	
	(0.353)		(0.367)		(0.022)	
Baseline standardized math score	0.231		0.286		0.055	
	(1.006)		(0.969)		(0.043)	
**Family:**						
One or both parents with ≥12 years of education (1 = yes)	0.202		0.223		0.021	
	(0.401)		(0.415)		(0.022)	
Family asset value, 10 thousand	2.836		3.305		0.469	
	(3.204)		(3.602)		(0.378)	
One or both parents out-migrated for work (1 = yes)	0.468		0.458		−0.010	
	(0.499)		(0.498)		(0.033)	
**Teacher & Schools:**						
Teachers finished college or higher education	0.501		0.512		0.011	
	(0.500)		(0.500)		(0.070)	
Share of teachers that held professional titles	0.945		0.847		−0.098 **	
	(0.288)		(0.199)		(0.037)	
Class size	44.232		45.237		1.005	
	(11.465)		(12.555)		(2.113)	
Half and above material taught on blackboard (1 = yes)	0.729		0.611		−0.118	
	(0.444)		(0.495)		(0.042)	
Number of observations	988		1099		-	

(**a**) Notes: Columns 1 to 2 and 4 to 5 present means with standard deviations reported in brackets. Columns 3 and 6 show coefficients estimated by regressing each variable on the treatment dummy, with standard errors, in parentheses, clustered at the school level. * *p* < 0.10. (**b**) Notes: Columns 1 to 2 present means with standard deviations reported in brackets. Column 3 shows coefficients estimated by regressing each variable on the treatment dummy, with standard errors, in parentheses, clustered at the school level. ** *p* < 0.05.

**Table 2:** Impact of providing eyeglasses on endline standardized mathematics score (ITT).

Dep. Var.: Endline Standardized Math Score	(1)	(2)	(3)	(4)
	Unadjusted	Adjusted	Unadjusted	Adjusted
Treatment (1 = free eyeglasses)	0.060 ***	0.062 ***	0.023	0.023
	(0.043)	(0.043)	(0.047)	(0.051)
Province (1 = Gansu)	−0.037	−0.050	−0.006	−0.059
	(0.146)	(0.160)	(0.152)	(0.170)
Treatment × Province (1 = Gansu)			0.077 **	0.081 **
			(0.089)	(0.088)
Treatment effect for Gansu			0.122 ***	0.123 ***
			(0.076)	(0.076)
Treatment effect for Shaanxi			0.023	0.031
			(0.047)	(0.048)
Baseline standardized math score controlled	Yes	Yes	Yes	Yes
student, family, teacher, characteristics controlled	-	Yes	-	Yes
Number of observations	2087	2087	2087	2087
R-square	0.397	0.408	0.399	0.411

Notes: Full sample is analyzed. Standardized coefficients are reported. Standard errors clustered at the school level are reported in parentheses. ** *p* < 0.05, *** *p* < 0.01.

**Table 3:** Compliance rate at baseline and endline.

Variable	Control Group	Treatment Group	Difference
	(1)	(2)	(3) = (2) − (1)
**Panel A. Gansu**			
Number of students with myopia	379	383	-
Wore eyeglasses at baseline (1 = yes)	0.113	0.141	0.027
	(0.317)	(0.348)	(0.024)
Wore eyeglasses at endline (1 = yes)	0.219	0.384	0.165 ***
	(0.414)	(0.487)	(0.033)
**Panel B. Shaanxi**			
Number of students with myopia	609	716	-
Wore eyeglasses at baseline (1 = yes)	0.166	0.170	0.004
	(0.372)	(0.376)	(0.021)
Wore eyeglasses at endline (1 = yes)	0.279	0.416	0.137 ***
	(0.449)	(0.493)	(0.026)

Note. Columns 1 to 2 present means with standard deviations reported in brackets. Column 3 shows coefficients estimated by regressing each variable on the treatment dummy, with standard errors, in parentheses, clustered at the school level. *** *p* < 0.01.

**Table 4:** Interaction effects of providing eyeglasses on the wearing of eyeglasses between provinces.

Dep. Var.: Endline Eyeglasses Wearing	(1)	(2)	(3)	(4)
	Unadjusted	Adjusted	Unadjusted	Adjusted
Treatment (1 = yes)	0.146 ***	0.140 ***	0.140 ***	0.132 ***
	(0.025)	(0.025)	(0.028)	(0.030)
Baseline eyeglass wearing (1 = yes)	0.336 ***	0.266 ***	0.336 ***	0.266 ***
	(0.028)	(0.034)	(0.028)	(0.034)
Province (1 = Gansu)	−0.066	−0.045	−0.073	−0.054
	(0.113)	(0.114)	(0.114)	(0.117)
Treatment × Province (1 = Gansu)			0.012	0.017
			(0.056)	(0.051)
Treatment effect for Gansu endline eyeglass wearing			0.159 ***	0.151 ***
			(0.049)	(0.042)
Treatment effect for Shaanxi endline eyeglass wearing			0.138 ***	0.132 ***
			(0.028)	(0.030)
Baseline standardized math score controlled	Yes	Yes	Yes	Yes
Student, family, teacher, characteristics controlled	-	Yes	-	Yes
Number of observations	2087	2087	2087	2087
R-square	0.213	0.252	0.213	0.252

Notes: Full sample is analyzed. Standardized coefficients are reported. Standard errors clustered at the school level are reported in parentheses. *** *p* < 0.01.

**Table 5:** Impact of wearing eyeglasses on endline standardized mathematics score (LATE).

Dep. Var.: Endline Standardized Math Score	Pooled Sample		Gansu		Shaanxi	
	Unadjusted	Adjusted	Unadjusted	Adjusted	Unadjusted	Adjusted
	(1)	(2)	(3)	(4)	(5)	(6)
Treatment (1 = yes)	0.861 ***	0.918 ***	1.536 **	1.770 ***	0.355	0.476
	(0.323)	(0.344)	(0.614)	(0.686)	(0.369)	(0.394)
Baseline standardized math score controlled	Yes	Yes	Yes	Yes	Yes	Yes
Student, family, teacher, characteristics controlled	-	Yes	-	Yes	-	Yes
R-square	0.258	0.258	−0.108	−0.123	0.393	0.367
Cragg–Donald Wald F statistic of the first stage	45.747	46.197	23.692	21.571	23.282	23.783
Number of observations	2087	2087	762	762	1325	1325

Notes: Full sample is analyzed. Standardized coefficients are reported. Standard errors clustered at the school level are reported in parentheses. ** *p* < 0.05, *** *p* < 0.01.

**Table 6:** Comparison of characteristics at baseline, by province.

Variables	Gansu	Shaanxi	Difference
	(1)	(2)	(3) = (2) − (1)
**Student:**			
Grade (1 = fourth)	4.622	4.591	−0.031
	(0.485)	(0.492)	(0.025)
Male sex (1 = yes)	0.517	0.484	−0.032
	(0.500)	(0.500)	(0.024)
Severity of myopia (Logmar-better eye)	0.495	0.522	0.056
	(0.249)	(0.220)	(0.015)
Not wearing eyeglasses at baseline (1 = yes)	0.873	0.832	−0.055 *
	(0.333)	(0.374)	(0.019)
Baseline standardized math score	0.143	0.327	0.090 **
	(0.971)	(0.990)	(0.070)
Math score below the average (1 = yes)	0.501	0.451	−0.049 **
	(0.500)	(0.498)	(0.023)
**Family:**			
One or both parents with high school education or higher (1 = yes)	0.235	0.201	−0.040
	(0.423)	(0.399)	(0.023)
One or both parents out-migrated for work (1 = yes)	0.524	0.428	−0.092 **
	(0.500)	(0.495)	(0.032)
Family asset value, 10 thousand	1.487	4.002	0.353 ***
	(1.899)	(3.757)	(0.238)
Household assets below the average (1 = yes)	0.927	0.606	−0.345 ***
	(0.261)	(0.489)	(0.019)
**Teacher & School:**			
Teachers with college or higher education (1 = yes)	0.487	0.518	0.030
	(0.500)	(0.500)	(0.070)
Share of teachers that held professional titles	0.912	0.883	−0.055
	(0.346)	(0.171)	(0.043)
Class size	47.189	43.365	−0.153
	(10.745)	(12.546)	(1.977)
Half and above material taught on blackboard (1 = yes)	0.831	0.565	−0.271 ***
	(0.375)	(0.496)	(0.062)
Number of observations	762	1325	-

Notes: Columns 1 to 2 present means with standard deviations reported in brackets. Column 3 shows standardized coefficients estimated by regressing each variable on the province dummy, with standard errors, in parentheses, clustered at the school level. * *p* < 0.10, ** *p* < 0.05, *** *p* < 0.01.

**Table 7:** Heterogeneity of ITT treatment effects by baseline characteristics.

Dep. Var.: Endline Standardized Math Score	Half and above Material Taught on Blackboard	Math Score below the Average	Household Asset below the Average	Parents Out-Migrated for Work	Not Wearing Eyeglasses at Baseline
	(1)	(2)	(3)	(4)	(5)
Treatment (1 = yes)	−0.029	0.033	−0.009	0.025	0.056
	(0.071)	(0.051)	(0.067)	(0.051)	(0.084)
Treatment × half and above material taught on blackboard	0.125 ***				
	(0.093)				
Half and above material taught on blackboard (1 = yes)	−0.100 ***				
	(0.069)				
Treatment × baseline standardized math below the average		0.052 *			
		(0.070)			
Baseline math score below the average (1 = yes)		0.051			
		(0.076)			
Treatment × household asset value below the average			0.095 **		
			(0.076)		
Household asset value below the average (1 = yes)			−0.097 *		
			(0.111)		
Treatment × one or both parents out-migrated for work				0.070 **	
				(0.066)	
One or both parents out-migrated for work (1 = yes)				−0.043 *	
				(0.046)	
Treatment × not wearing eyeglasses at baseline					0.007
					(0.088)
Not wearing eyeglasses at baseline (1 = yes)					−0.019
					(0.069)
Treatment effect for subgroup if dummy equals one	0.098 ***	0.081 ***	0. 090 ***	0.112 ***	0.062 ***
	(0.054)	(0.062)	(0.048)	(0.058)	(0.046)
Treatment effect for subgroup if dummy equals zero	−0.032	0.038	−0.028	0.032	0.050
	(0.071)	(0.051)	(0.067)	(0.051)	(0.084)
Baseline standardized math score controlled	Yes	Yes	Yes	Yes	Yes
Student, family, teacher, characteristics controlled	Yes	Yes	Yes	Yes	Yes
R-square	0.411	0.411	0.411	0.410	0.408
Number of observations	2087	2087	2087	2087	2087

Notes: Full sample is analyzed. Standardized coefficients are reported. Standard errors clustered at the school level are reported in parentheses. * *p* < 0.10, ** *p* < 0.05, *** *p* < 0.01.

**Table 8:** Explained percentage of the total difference in ITT impact by baseline characteristics.

Dep. Var.: Endline Standardized Math Score	Half and above Material Taught on Blackboard	Math Score below the Average	Household Asset below the Average	Parents Out-Migrated for Work
	(1)	(2)	(3)	(4)
Coefficients of difference term (As shown in Table 6)	−0.271	−0.049	−0.345	−0.092
Coefficients of interaction term (As shown in Table 7)	0.100	0.052	0.095	0.070
Coefficients of interaction term × coefficients of difference term (SD)	−0.027	−0.003	−0.033	−0.006
Explained percentage of the total difference in ITT impact (%)	29.45	3.26	35.87	6.52

## Data Availability

The datasets used and analyzed during the current study are available from the corresponding author on reasonable request.

## Appendix A

**Table A1:** Baseline characteristics difference between attrition and no missing sample.

Variables	Pooled Sample (*n* = 2169)			Gansu (*n* = 786)			Shaanxi (*n* = 1383)		
	Attrition Group	None Attrition Group	Difference	Attrition Group	None Attrition Group	Difference	Attrition Group	None Attrition Group	Difference
	(1)	(2)	(2) − (1)	(4)	(5)	(5) − (4)	(7)	(8)	(8) − (7)
**Student:**									
Grade (1 = fourth)	4.644	4.6	−0.044	4.75	4.622	0.128	4.603	4.591	−0.012
	(0.481)	(0.49)	(0.057)	(0.442)	(0.485)	(0.092)	(0.493)	(0.492)	(0.069)
Male Sex (1 = Yes)	0.415	0.496	0.081	0.5	0.517	−0.017	0.379	0.484	0.105
	(0.496)	(0.500)	(0.058)	(0.511)	(0.500)	(0.092)	(0.489)	(0.500)	(0.072)
Severity of Myopia	0.514	0.512	−0.002	0.543	0.495	−0.048	0.502	0.522	0.02
	(0.255)	(0.231)	(0.028)	(0.292)	(0.249)	(0.058)	(0.24)	(0.22)	(0.031)
Not Wearing Eyeglasses at Baseline (1 = Yes)	0.854	0.847	−0.007	0.833	0.873	−0.04	0.862	0.832	−0.03
	(0.356)	(0.36)	(0.036)	(0.381)	(0.334)	(0.069)	(0.348)	(0.374)	(0.043)
Baseline standardized math score	0.071	0.260	0.189	−0.286	0.143	0.429 *	0.218	0.328	−0.189
	(1.152)	(0.987)	(0.124)	(1.187)	(0.971)	(0.216)	(0.146)	(0.027)	(0.123)
**Family:**									
One or Both Parents with ≥12 years education (1 = Yes)	0.293	0.213	−0.08	0.333	0.235	−0.098	0.276	0.201	−0.075
	(0.458)	(0.408)	(0.048)	(0.482)	(0.423)	(0.09)	(0.451)	(0.399)	(0.057)
Family Asset Value, 10 thousand	2.926	3.083	0.157	1.793	1.487	−0.306	3.395	4.002	0.607
	(3.52)	(3.426)	(0.419)	(2.842)	(1.899)	(0.539)	(3.686)	(3.757)	(0.528)
One or Both parents out-migrated for work (1 = Yes)	0.549	0.463	−0.086	0.625	0.524	−0.101	0.517	0.428	−0.089
	(0.5)	(0.499)	(0.055)	(0.495)	(0.5)	(0.09)	(0.504)	(0.495)	(0.069)
**Teacher & Schools:**									
Teachers Finished College or Higher Education	0.488	0.507	0.019	0.458	0.487	0.029	0.500	0.518	0.018
	(0.503)	(0.500)	(0.064)	(0.509)	(0.5)	(0.12)	(0.504)	(0.5)	(0.075)
Share of Teachers that Held Professional Titles	0.876	0.893	0.017	0.908	0.912	0.004	0.863	0.883	0.02
	(0.182)	(0.25)	(0.022)	(0.154)	(0.346)	(0.041)	(0.192)	(0.171)	(0.023)
Class Size	42.439	44.761	2.322	43.75	47.189	3.439	41.897	43.365	1.469
	(13.006)	(12.059)	(1.62)	(12.333)	(10.745)	(3.17)	(13.341)	(12.546)	(1.938)
Observations	82	2087	-	24	762	-	58	1325	-

Notes: Columns 1 to 3 show the descriptive statistics and attrition check for the whole pooled sample. Columns 4 to 6 show the descriptive statistics and attrition check for Gansu province. Columns 7 to 9 show the descriptive statistics and attrition check for Shaanxi province. Information on blackboard use was missing for 80 of the 82 attrition sample and therefore was not shown. The coefficients of difference estimated by regressing each of the baseline characteristics on the attrition dummy with standard errors clustered at the school level in parentheses. * *p* < 0.10.
